# Inhibition of NADPH Oxidases Activity by Diphenyleneiodonium Chloride as a Mechanism of Senescence Induction in Human Cancer Cells

**DOI:** 10.3390/antiox9121248

**Published:** 2020-12-08

**Authors:** Katarzyna Piszczatowska, Dorota Przybylska, Ewa Sikora, Grażyna Mosieniak

**Affiliations:** 1 Laboratory of Molecular Bases of Aging, Nencki Institute of Experimental Biology of Polish Academy of Sciences, Pasteura 3 St., 02-093 Warsaw, Poland; kpiszczatowska@wum.edu.pl (K.P.); dprzybylska87@gmail.com (D.P.); e.sikora@nencki.edu.pl (E.S.); 2 Chair and Department of Biochemistry, Medical University of Warsaw, Banacha 1 St., 02-097 Warsaw, Poland

**Keywords:** senescence, apoptosis, cancer, ROS, NADPH oxidases, DPI

## Abstract

NADPH oxidases (NOX) are commonly expressed ROS-producing enzymes that participate in the regulation of many signaling pathways, which influence cell metabolism, survival, and proliferation. Due to their high expression in several different types of cancer it was postulated that NOX promote tumor progression, growth, and survival. Thus, the inhibition of NOX activity was considered to have therapeutic potential. One of the possible outcomes of anticancer therapy, which has recently gained much interest, is cancer cell senescence. The induction of senescence leads to prolonged inhibition of proliferation and contributes to tumor growth restriction. The aim of our studies was to investigate the influence of low, non-toxic doses of diphenyleneiodonium chloride (DPI), a potent inhibitor of flavoenzymes including NADPH oxidases, on p53-proficient and p53-deficient HCT116 human colon cancer cells and MCF-7 breast cancer cells. We demonstrated that the temporal treatment of HCT116 and MCF-7 cancer cells (both p53 wild-type) with DPI caused induction of senescence, that was correlated with decreased level of ROS and upregulation of p53/p21 proteins. On the contrary, in the case of p53−/− HCT116 cells, apoptosis was shown to be the prevailing effect of DPI treatment. Thus, our studies provided a proof that inhibiting ROS production, and by this means influencing ROS sensitive pathways, remains an alternative strategy to facilitate so called therapy-induced senescence in cancers.

## 1. Introduction

The role of oxidative stress in cellular transformation was postulated many years ago leading to the so called “free radical theory of cancer”. The seminal paper by Oberley and colleagues presented the idea that reactive oxygen species act as second messengers and stimulate cell proliferation but could also activate a cascade of molecular events that could lead to cell immortality [1]. Since that time, a considerable experimental support has accumulated proving that reactive oxygen species (ROS) participate in transformation and cancer development in many ways.

Increased ROS production can lead to direct damage of biomolecules, such as nucleic acids, and result in genomic instability, which is associated with development of many pathologies including cancer. ROS can also modulate redox-sensitive signaling pathways leading to their activation or inhibition. Moreover, the modification of proteins and lipids by ROS might influence cell functioning and, in consequence, promote uncontrolled cell growth and cell transformation [2].

The main intracellular source of ROS is mitochondrial electron transfer chain but also a family of enzymes specialized for ROS production called NADPH oxidases (NOX). So far seven membrane-bound NOX isoforms, referred to as NOX1 to NOX5, dual oxidase 1 (DUOX1) and 2 (DUOX2), have been identified. Despite marked similarities they differ in structure, biochemical features, and subcellular localization. NOX enzymes are localized at the membranes of different organelles where they regulate redox signaling pathways [3].

Importantly, NOX catalytic and regulatory subunits have been identified in several of the most common cancer types. Increased expression of NOX1, NOX2, NOX4, and NOX5 or their regulatory components has been shown in various cancer cell lines or human tumors when comparing early and late stages of tumorigenesis [4,5,6,7,8,9,10,11,12,13,14,15].

Several molecular mechanisms by which increased activity of NOX can support tumor growth, development, and metastasis, have been described. It was demonstrated that ROS produced by NOX lead to tyrosine phosphatases inactivation. As a result, receptor tyrosine kinases, which undergo autophosphorylation upon growth factor stimulation, remain active, causing the induction of downstream signaling pathways [16,17,18]. Indeed, survival pathways regulated by Janus kinase (JAK)–signal transducer and activator of transcription (STAT), AKT, nuclear factor kappa B (NF-κB), and p53 signaling were shown to be activated by ROS produced by NADPH oxidases [19,20]. Moreover, pro-apoptotic genes expression regulated by p53 was shown to be inhibited by NOX1derived ROS. The observed effect resulted from ROS-induced activation of sirtuin 1 (SIRT1) and subsequent deacetylation and inactivation of p53 [21,22].

It was demonstrated that ROS produced by NOX1 and NOX4 inhibit activity of PTEN phosphate [23]. Consequently, NOX can actively regulate the PI3K-AKT-mTOR signaling pathway and, by this means, promote mRNA translation, cell survival, and metabolism, which were shown to be critical for different types of cancer [24,25,26]. Moreover, ROS produced by NOX1 and NOX4 were shown to foster vascularization of prostate cancer in a mouse model of fibrosarcoma via inducing the expression of VEGF and VEGFR [27].

Thus, due to its pleiotropic and protumorigenic activity, ROS-producing enzymes have been considered as an important target for anticancer therapy. One of the possible molecular mechanisms that has already been shown to support cancer treatment is cellular senescence. Many in vitro and in vivo studies have proved that chemo- or radiotherapy leads to the induction of growth arrest of cancer cells, a phenomenon called therapy-induced senescence [28,29,30,31]. The main trigger of senescence is induction of double-strand DNA damage that activates the p53-dependent signaling pathway, which leads to cell cycle arrest but also significant changes in cell morphology and metabolism [32,33,34]. Induction of cancer cells senescence can restrict tumor growth but also, due to activation of immune cells by factors secreted by senescent cells, may lead to regression of cancer [35].

Taking into account the important role of NOX in supporting tumor growth, we hypothesize that temporal inhibition of ROS-producing enzymes might lead to prolonged growth arrest of cancer cells. To this end we tested the influence of diphenyleneiodonium (DPI) on p53 proficient and p53 deficient colon cancer cells and breast cancer cells. DPI inhibits the activity of flavoenzymes, thus it cannot be treated as a specific NOX family inhibitor and the effect of its activity must be interpreted with certain caution. However, recently, it has been demonstrated that among several different NOX inhibitors, DPI was the most effective in inhibiting all NOX isoforms. It thus meets the criteria of a bona fide NOX inhibitor [36]. Our studies revealed that decreased ROS production due to DPI treatment induces senescence in cancer cells in a p53 dependent manner while lack of p53 expression results in apoptosis. This indicates that NOX inhibition may exert anticancer effect on both wild type p53 expressing cancer cells and cancer cells with mutated p53 but through a different mechanism.

## 2. Materials and Methods

### 2.1. Cell Lines/Cell Culture and Treatments

The human colon HCT116 (p53+/+ and p53−/−) cancer cell line was kindly provided by Dr. Bert Vogelstein (Johns Hopkins University, Baltimore, MD, USA), MCF-7 breast cancer cell line were purchased from American Type Culture Collection, ATCC (LGC, Warsaw, Poland). HCT116 p53+/+ and p53−/− cells were cultured in McCoy’s medium, MCF-7 cells were cultured in DMEM; both media were supplemented with 10% fetal bovine serum (FBS) and antibiotics. The cells were cultured at a density of 10^4^ cells/cm^2^. When the cells were treated with DPI (Sigma-Aldrich, Saint Louis, MO, USA), DMSO was added to control cells to test the effect of the solvent.

### 2.2. Estimation of Cell Viability by MTT Assay

To evaluate cell viability of DPI treated cells or controls in selected time points, a solution of 3-(4,5-Dimethylthiazol-2-yl)-2,5-diphenyltetrazolium bromide (MTT), at a concentration of 5 mg/mL (Sigma-Aldrich), was added to cells seeded 24 h before DPI treatment in a 96-well plate, at the density of 2000 cells per well. Incubation with MTT was performed for 2 h in a humidified atmosphere (5% CO_2_) at 37 °C and dimethyl sulfoxide (DMSO) (Sigma-Aldrich) was used to dissolve formazan formed in metabolically active living cells. The absorbance of the samples was measured at the wavelength of 570 nm with a microplate reader (Reader 400 SFC, LabInstruments, Hamburg, Germany).

### 2.3. DNA Content and Cell Cycle Analysis

The cells were collected at indicated time points for DNA analysis. To this aim, they were fixed in the 70% ethanol and stained with propidium iodide (PI) solution containing 50 μg/mL PI in PBS, 3.8 mM sodium citrate, 500 μg/mL RNase (all agents from Sigma-Aldrich). The amount of DNA was assessed using a flow cytometer (FACSCalibur, BD Biosciences, Franklin Lakes, NJ, USA) with CellQuest^TM^Pro Software (version 6, BD Biosciences, Franklin Lakes, NJ, USA). A total of 10,000 events were collected for each sample.

### 2.4. Western Blotting Analysis

The preparation of whole cell protein extracts was performed with SLB buffer (10% glycerol, 2% SDS, 50 mM Tris-HCl pH 6.8) and the Bicinchoninic Acid kit (BCA) was used for protein determination according to the manufacturer’s protocol (Sigma-Aldrich). Proteins (20 μg per well) were separated during electrophoresis in SDS polyacrylamide gels and then transferred to nitrocellulose membranes. Afterwards, membranes have been placed in solution of 5% non-fat milk dissolved in TBST (TBS with 0.1% Tween-20 from Sigma-Aldrich) and incubated at room temperature (RT) for 1 h. Thereafter, membranes were incubated with monoclonal or polyclonal primary antibodies in appropriate concentrations: anti-p21(C-19) (1:500), anti-p53 (DO-1) (1:500) (Santa Cruz Biotechnology Inc., Dallas, TX, USA), anti p-p53 (Ser15)(1:500), anti-γH2AX (1:500) (Cell Signaling Technology, Danvers, MA, USA), anti-GAPDH (1:50,000) (Merck Millipore, Burlington, MA, USA), anti-PARP1 (1:1000) (Becton Dickinson, Franklin Lakes, NJ, USA). Secondary antibodies conjugated with horseradish peroxidase and the ECL reagents kit (Thermo Fisher Scientific, Waltham, MA, USA) were applied, according to the manufacturer’s protocol, to detect proteins.

### 2.5. Cytochemical Detection of SA-β-Gal

SA-β-Gal activity detection assay was carried out according to Dimri et al. [37]. At appropriate time points, the cells were fixed in 0.2% glutaraldehyde in PBS, 2% formaldehyde, and then washed and incubated overnight at 37 °C with a mixture of 5 mM potassium ferrocyanide, 150 mM NaCl, 2 mM MgCl_2_, 0.02 M phosphate buffer, pH 6.0 and 1 mg/mL 5-bromo-4-chloro-3-indolyl-b-d-galactopyranoside (all agents from Sigma-Aldrich). Results were visualized using a Nikon Eclipse Ti-U microscope and Nikon Digital Sight DS-U3 camera (Nikon, Tokyo, Japan) images were taken in a transmitted light.

### 2.6. Bromodeoxyuridine Incorporation Assay

The cells were seeded on coverslips and, at indicated time points, used for DNA synthesis assay. To this aim, 10 μM bromodeoxyuridine (BrdU, Sigma-Aldrich) was added to the cell culture medium for 24 h. Afterwards, 70% ethanol was used for fixation and cells were stored at −20 °C overnight. The detection of BrdU was performed using primary antibody directed against BrdU (Becton Dickinson) and, in the next step, a secondary antibody, that is, Alexa 488-conjugated IgG (Becton Dickinson). The cells were examined under a fluorescence microscope at 450–490 nm excitation wavelength (Nikon, Tokyo, Japan) and images were documented with an Evolutions VF digital CCD camera (MediaCybernetics, Rockville, MD, USA). An analysis of at least 200 cells was conducted in each experiment and the percentage of BrdU-positive cells was calculated.

### 2.7. ROS Content Analysis

For analysis of ROS level, cells were collected at indicated time points and counted. Afterwards, the same number of cells—approximately 100,000, were incubated in 37 °C, 5% CO_2_ for 30 min with 10 μM carboxy-H_2_DCFDA (6-carboxy-2′,7′-dichlorodihydrofluorescein diacetate; Invitrogen) in serum-free medium. Carboxy-H_2_DCFDA is widely used as an indicator of general oxidative stress. In the next step, PBS was used to wash cells, then cells were incubated in serum-free medium with probe. Analysis of samples was performed immediately after incubation with the use of a flow cytometer (Becton Dickinson) with FL-2 channel and 30,000 events were collected. CellQuest Pro software was applied for analysis.

### 2.8. Statistical Analysis

Data were analyzed using GraphPad Prism Software. To evaluate statistical significance of MTT test one-way ANOVA with post-hoc Tukey test was used. In all other analysis data were subjected to *t*-Test analysis. The *p*-value was marked as follows: * *p* < 0.05, ** *p* ≤ 0.01; *** *p* ≤ 0.001.

## 3. Results

**Figure 1:**
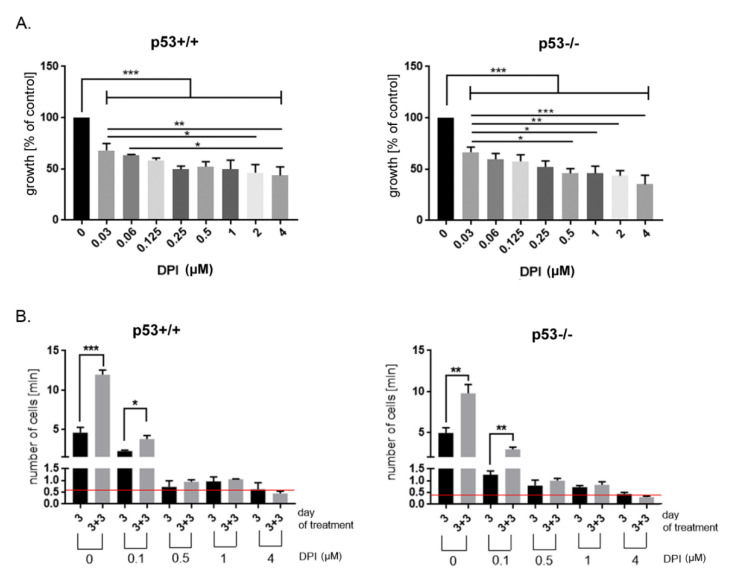
Diphenyleneiodonium chloride (DPI) exerts growth inhibitory effect in HCT116 p53+/+ and p53−/− cancer cells. (**A**) The influence of different concentrations of DPI on HCT116 cell viability after 24 h of treatment (MTT assay); (**B**) Inhibition of cell proliferation upon DPI treatment. HCT116 cells were treated with different concentrations of DPI and cells were counted after 3 days of treatment and after subsequent 3 days of culture in DPI-free medium (day 3 + 3). Red lines mark the initial number of cells, * *p* < 0.05, ** *p* ≤ 0.01; *** *p* ≤ 0.001.

In order to estimate the dose dependent effect of DPI on cancer cells, we treated HCT116 p53+/+ and p53−/− cells with different concentrations of the inhibitor and performed MTT assay after 24 h of treatment. MTT assay is based on measurement of metabolic activity of the cells and indirectly enables to estimate changes in the number of treated cells comparing to control, untreated ones. The analysis revealed that DPI applied in a low, nanomolar concentration significantly decrease HCT116 cells growth. Moreover, the response was partially concentration-dependent only when tested in p53−/− cells while HCT116 p53+/+ showed similar sensitivity to the drug treatment at concentration range between 0.125 and 4 µM. This result suggests that the observed effect was mainly cytostatic without pronounced toxicity. The sensitivity of p53 proficient and p53 deficient cells was very similar and no statistically significant differences between p53+/+ and p53−/− cells were revealed at either concentration of DPI (Figure 1A).

Accordingly, we decided to test the effect of prolonged treatment of cancer cells with selected concentrations of DPI. To this end cells were cultured in the presence of DPI for 3 days, then left in inhibitor-free medium for subsequent 3 days and counted. We observed a growth inhibitory effect of DPI, which was dose-dependent (Figure 1B). The lowest concentration (100 nM) slowed down the proliferation of cells but did not arrested them, since after 3 days of culture in the presence of DPI the number of cells was higher than in the initial cell culture (the number of cells at day 0 marked as a red line). The removal of DPI-containing medium led to regrowth of cells, the number of which increased significantly comparing to DPI-treated culture (day 3 versus day 3 + 3). DPI used in concentrations equal or higher than 500 nM caused more pronounced growth arrest which lasted for subsequent days even though cells were cultured in inhibitor-free medium. The prolonged culture of cancer cells with higher concentration of DPI (4 µM) entailed a visible toxic effect since the number of cells at day 3 + 3 dropped below the initial cell number. Interestingly, no remarkable differences were observed between p53+/+ and p53−/− cells when comparing both the concentration-dependent response and the extent of growth inhibition potential of DPI.

**Figure 2:**
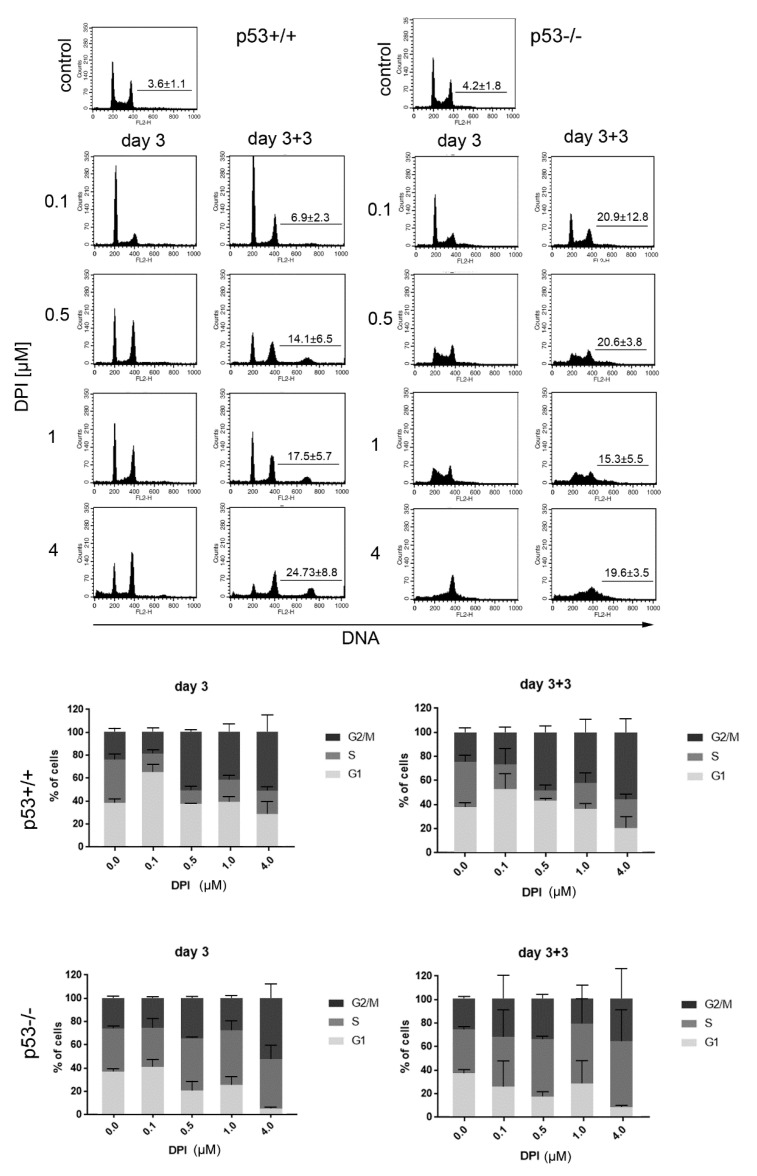
DPI disturbs cell cycle progression in HCT116 p53+/+ and p53−/− cells. HCT116 cells were treated with different concentrations of DPI and cell cycle distribution was analyzed 3 days after treatment and after subsequent culture in DPI-free medium. Representative histograms (upper panel) and graphs presenting distribution of cells in different phases of cell cycle. Mean values from 4 independent experiments ±SD. The mean number (±SD) of polyploid cells is marked on each histogram.

In order to perform an in-depth analysis of DPI influence on cancer cell proliferation we performed cell cycle analysis at day 3 and day 3 + 3 (Figure 2). Substantial differences between p53 proficient and p53 deficient cells were revealed in cell cycle distribution. Cells expressing p53 and treated with DPI showed decreased percentage of cells in the S phase comparing to control. DPI, at 100 nM concentration, caused accumulation of cells in the G1 phase and a decrease in the number of G2/M cells. Cells treated with higher concentration of DPI (0.5, 1, and 4 µM) were arrested in G1 and G2/M phases. Importantly, the cell cycle arrest persisted for the subsequent 3 days of culture in the absence of DPI, although a gradual increase in polyploid cell fraction was also observed in a concentration-dependent manner.

On the contrary, cells that lack p53 could not be arrested in cell cycle for a longer time. Instead, a decrease in G1 and increase in S phase cells was observed upon treatment with 0.5–4 µM DPI. The high number of cells with DNA content between 2N and 4N reflected gradual loss of DNA in G2/M phase cells due to cell death rather than increased number of cells able to synthesize DNA. Changes observed after 3 days of treatment did not revert even after a 3 day culture in DPI-free medium.

**Figure 3:**
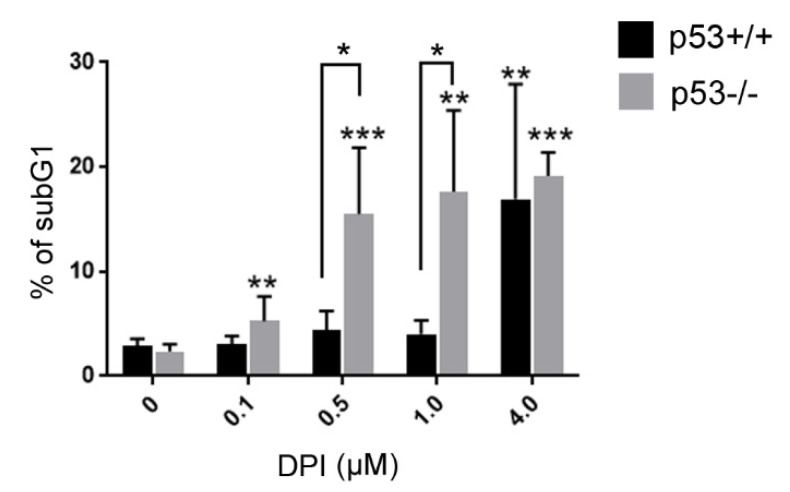
DPI-treatment induces DNA fragmentation preferentially in HCT116 p53−/− cells. HCT116 cells were treated with different concentrations of DPI for 3 days and subsequently cultured in DPI-free medium for 3 days. SubG1 fraction was measured based on flow cytometry analysis of DNA content. Mean ± SD of the percentage of cells in the subG1 fraction, measured in at least 3 independent experiments, is presented, * *p* < 0.05, ** *p* ≤ 0.01; *** *p* ≤ 0.001.

Moreover, we analyzed the extent of apoptosis induction by estimating the subG1 fraction in DPI treated p53+/+ and p53−/− cells. The subG1 fraction represents cells with fragmented DNA, which is removed from the cell upon the staining procedure. As a result, 2N cells that underwent apoptotic DNA fragmentation constitute a subG1 population. We have noticed marked differences between p53+/+ and p53−/− cells. P53−/− cells treated with even the lowest DPI concentration showed significantly increased subG1 fraction and the amount of those cells increased further in a concentration dependent manner. On the contrary, a pronounced increase in subG1 p53+/+ cells was observed only after treatment with the highest DPI concentration. Significant differences between p53 positive and negative cells were observed upon 0.5 and 1 µM DPI treatment (Figure 3).

**Figure 4:**
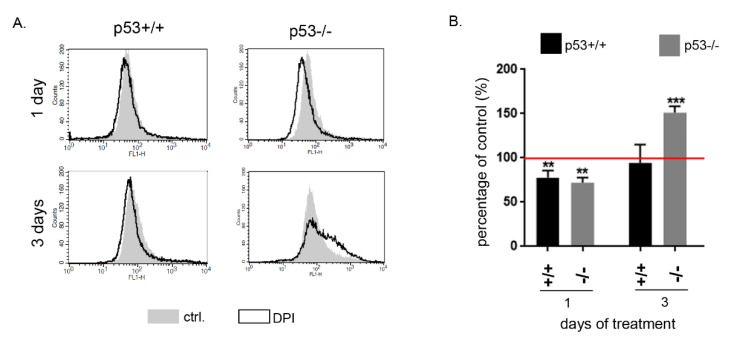
DPI decreases ROS level in HCT116 p53 proficient and p53 deficient cells. Cells were treated with 0.5 µM DPI and after 1 or 3 days the level of ROS was estimated using the H_2_DCFDA probe. (**A**) Representative histograms and relative ROS level in DPI treated cells are presented (**B**) Mean ROS level ±SD from 4 independent experiments; ** *p* ≤ 0.01; *** *p* ≤ 0.001.

To reveal how changes observed in the cell cycle correlate with ROS level we performed cytometric analysis using a ROS-sensitive fluorescence probe. We treated p53+/+ and p53−/− HCT116 cells with 0.5 µM DPI. This concentration was the lowest one that induced a prolonged growth arrest of the cells. After 24-h treatment we observed a significant decrease in ROS level in both clones. This effect was observed also after 3 days of treatment in p53+/+ cells while a significant increase in ROS was detected in p53−/− cells (Figure 4). This upregulation of ROS production in p53 deficient cells correlates with increased cell death at a later time point as shown by cell cycle analysis (Figure 2 and Figure 3).

**Figure 5:**
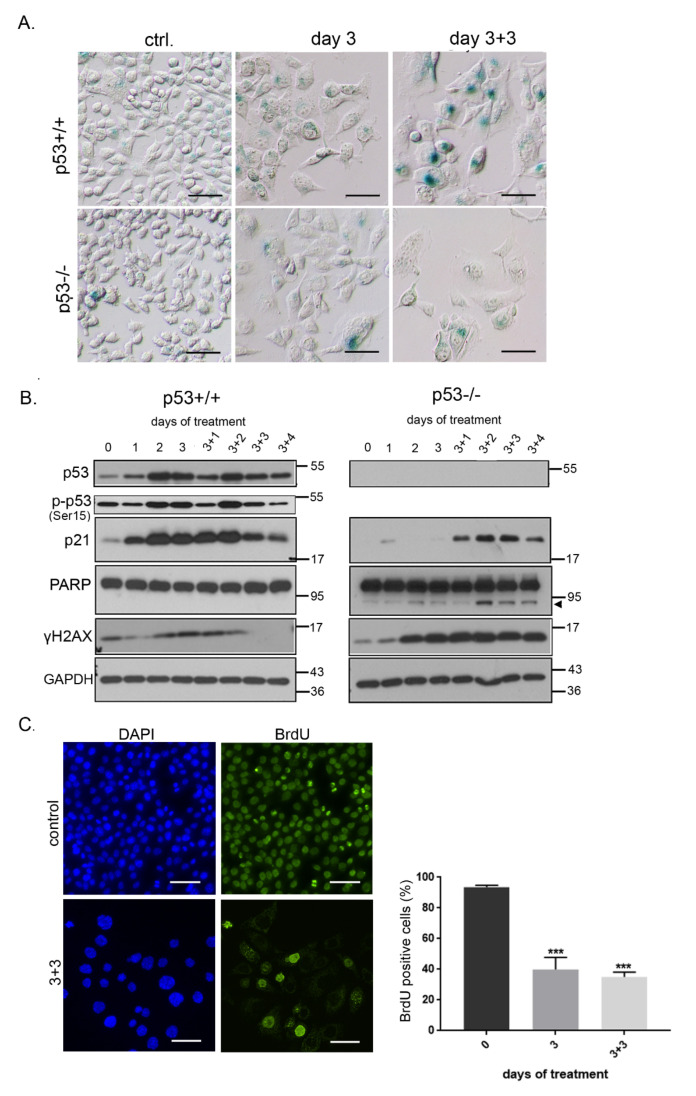
DPI treatment induces senescence of HCT116 cells. (**A**) HCT116 cells were treated with 0.5 µM DPI for 3 days and SA-β-gal activity was analyzed; (**B**) the level of protein markers of senescence—p53, p-p53 and p21, and apoptosis—PARP cleavage (indicated by arrowhead) and γH2AX—was estimated at different time points after DPI (0.5 µM) treatment. (**C**) BrdU incorporation analysis was performed in HCT116 p53+/+ cells. The percentage of cells that were able to incorporate BrdU within 24 h of culture is presented on the graph (mean ± SD, four experiments); *** *p* ≤ 0.001.

To finally reveal the cause of prolonged growth inhibition of DPI treated cells we analyzed the expression of senescence markers in HCT116 cells. SA-β gal analysis proved induction of senescence upon DPI treatment. The level of SA-β gal activity increased with time (increased intensity of blue staining) resulting in gradual development of the senescent phenotype. Of note, the part of population of p53−/− that survived DPI treatment also underwent senescence, however, the number of SA-β gal positive cells and the level of its activity was lower than in p53+/+ cells (Figure 5A). The upregulation of p53 and its phosphorylated form (Ser15), and of its downstream target—p21 protein in p53 proficient cancer cells confirmed the induction of senescence. In contrast to p53+/+ cells, induction of p21 was weaker and postponed in time in p53−/− cells, which correlated with lower level of SA-β gal activity. Partial cleavage of Poly (ADP-ribose) polymerase (PARP) by apoptotic caspases, was detected only in p53−/− cells. It correlates with the results of DNA content analysis, which demonstrated an increased fraction of p53−/− cells in subG1. Moreover, we noticed a high level of γH2AX histone exclusively in p53−/− cells. This phosphorylation of a core histone marks the site of double strand DNA damage. The level of γH2AX increases during apoptosis simultaneously with the formation of high molecular weight DNA fragments [38]. Altogether, these results prove that DPI induces apoptosis in p53-deficient cells (Figure 5B). BrdU incorporation test confirmed that more than 60% of p53+/+ cells were not able to synthesize DNA. The number of BrdU negative cells did not change even after culturing in DPI-free medium suggesting that they underwent a prolonged growth arrest (Figure 5C).

**Figure 6:**
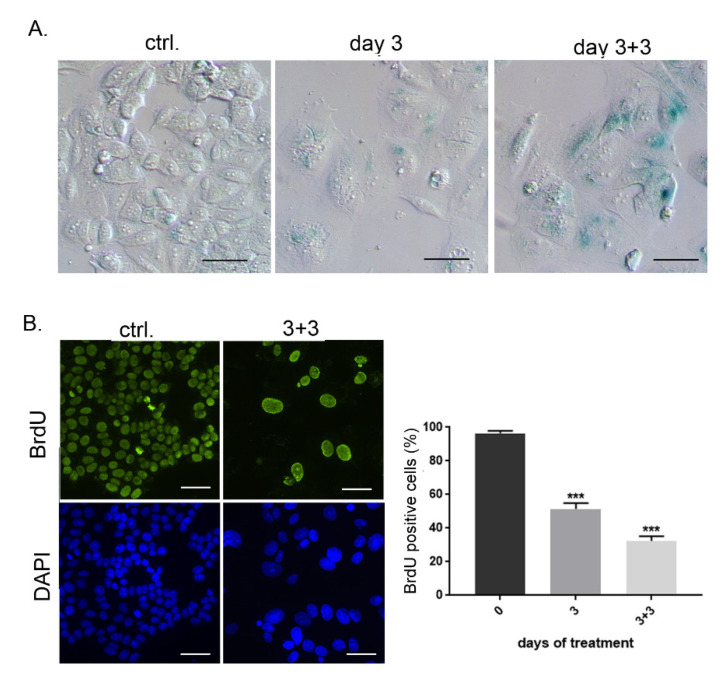
DPI treatment induces senescence of MCF-7 cells. (**A**) Breast cancer MCF-7 were treated with 0.5 µM DPI for 3 days and SA-β-gal activity was analyzed at indicated time points; (**B**) BrdU incorporation test was performed in untreated and DPI-treated cells and the percentage of cells that were able to incorporate BrdU within 24 h of culture is presented on the graph (mean ± SD, three experiments); *** *p* ≤ 0.001.

Additionally, we analyzed the influence of DPI on human breast cancer MCF-7 cells and revealed that DPI treatment also led to senescence induction in these cells (Figure 6), suggesting that inhibition of ROS production may exert a prolonged growth arrest in cancer cells of different origin.

## 4. Discussion

Reactive oxygen species, which participate in many different signaling pathways, play a role in the regulation of cell survival and proliferation. This aspect of redox biology is particularly important in the context of cancer development and progression. Our results have demonstrated for the first time that downregulation of ROS level by treatment of cancer cells with an inhibitor of flavin—containing enzymes—DPI, leads to prolonged growth arrest of cancer cells that correlates with senescence induction. Since cellular senescence has already been shown to exert a beneficial role in cancer treatment, we postulate that, apart from well-known DNA damaging chemotherapeutic drugs, also inhibitors of ROS producing enzymes might be considered potentially useful for cancer therapy.

We showed that treatment of HCT116 p53+/+ and p53−/− cells with DPI for 24 h led to growth arrest in a wide concentration range of DPI. Moreover, there were no significant differences between p53 proficient and deficient cells indicating that the growth inhibitory effect of DPI was p53 independent. Similar results were presented by Song et al. (2008) [39] who revealed that DPI inhibited the proliferation of HCT116 (wild type p53) and HL-60 (p53 null) cancer cells without significant influence on cell viability. DPI induced cell cycle arrest relied on p53/p21 induction in HCT116 cells while the downregulation of cyclin D1 was responsible for similar effect in p53-deficient HL-60 cell line [36]. The growth inhibitory effect of DPI treatment was also demonstrated for other colon cancer cell lines (Caco2, HT-29, LS-174T) at a concentration range 10–250 nM. Importantly, further studies have shown that DPI, at doses that produce plasma concentrations corresponding to those used in in vitro experiments, inhibits the growth of tumor xenografts of two human colon cancer cell lines [40].

Although short term growth arrest upon DPI treatment was p53-independent, prolonged (3 day) exposure of HCT116 cells to DPI revealed a pronounced difference between p53+/+ and p53−/− cells. Cell cycle analysis has proved that p53 expressing cells were arrested in the G1 and G2/M phase of cell cycle even after a subsequent 3-day culture in DPI-free medium. On the other hand, DNA fragmentation and PARP cleavage was induced in p53 deficient HCT116 cells treated with DPI concentration higher than 100 nM. Thus, we conclude that p53 expression facilitates a prolonged cell cycle arrest and protects cells from apoptosis induction upon DPI treatment. We made a similar observation when HCT116 cells (both p53+/+ and p53−/−) were treated with curcumin, a natural polyphenol with known anticancer properties [41]. On the contrary, Park and coworkers [42] revealed that the induction of p53 in DPI treated RPE cells led to apoptotic cell death that was preceded by an increase in the level of proapoptotic and a decrease in the level of antiapoptotic members of Bcl-2 family. Of note, DPI-induced apoptosis in RPE cells was detected upon treatment with relatively high doses of the inhibitor (10 µM) comparing to concentration used in our studies. Indeed, using 4 µM DPI for 3 days, we detected a higher number of subG1 cells in HCT116 p53+/+ cells. Thus, we cannot rule out the possibility that higher doses of DPI induce cell death due to the proapoptotic activity of p53.

Our studies showing DPI-induced G1 and G2/M arrest are consistent with the results of Song et al. (2008) and Venkatachalam et al. (2008) [39,43], indicating that this inhibitor blocks progression from the G1 to S phase leading to cell accumulation in G1 [40]. DPI also affects the G2/M checkpoint either by postponing entry into mitosis [43] or by inducing premature exit from this phase of cell cycle [44]. In any case accumulation of 4N DNA cells was observed. Interestingly, DPI-treated cells that were not able to finalize mitosis remained in the interphase and did not resume proliferation.

The morphological observation of DPI-treated HCT116 cells demonstrated that cells became bigger and flatter what is typical for senescence. Indeed, analysis of SA-β-gal activity proved that prolonged growth arrest induced by DPI correlates with appearance of SA-β-gal—positive cells particularly among HCT116 p53+/+ cells. Expression of SA-β-gal was accompanied by significant reduction of the number of cells that were able to incorporate BrdU, which was already observed after 3 days of DPI treatment and lasted through the subsequent 3 days of culture in DPI-free medium. Moreover, prolonged activation of the p53/p21 pathway was also observed. Altogether, we have proved that temporal treatment of cancer cells with DPI leads to senescence induction. Senescence induction was also observed in DPI-treated breast cancer MCF-7 cells. Interestingly, the appearance of SA-β-gal—positive HCT116 p53−/− cells as well as a low level of p53-independent induction of p21 was also observed, which indicates that p53 protein is indispensable for cancer cell senescence. This observation is consistent with our previous studies in which we induced senescence with chemotherapeutic drug—doxorubicin [45]. However, in the case of p53 deficient cells, apart from senescence induction we have observed apoptosis, which proves that p53 induction is necessary for survival of growth arrested cells. The treatment of cells with low concentration of DPI that induces senescence, led to significant reduction of ROS level indicating that inhibition of ROS producing enzymes might be responsible for the permanent growth arrest of cancer cells. Recently, we have demonstrated that selective downregulation of NOX4 in vascular smooth muscle cells resulted in senescence induction in those cells [46]. These results further indicate that we may induce prolonged inhibition of cell proliferation just by influencing ROS-sensitive signaling pathway. So far reports concerning the influence of DPI effect on ROS level were contradictory. It was demonstrated that DPI might induce oxidative stress depending on concentration, cell type and time of exposure [47,48]. Indeed, we noticed upregulation of ROS level in p53−/− cells but only after 3 days of treatment. We hypothesize that this might be related to apoptosis induction that was observed only in cells lacking p53, since increased ROS production often correlates with late phase of apoptosis and contributes to cell death.

Recently, it was demonstrated that the treatment of mesenchymal stem cells with different antioxidants leads also to permanent growth arrest of these cells [49]. Of note, antioxidants induced ϒH2AX expression, which is indicative of DNA damage. They hypothesized that it resulted from replication stress and/or impaired ability of DNA repair upon antioxidant treatment. On the contrary, we did not observe increased phosphorylation of histone γH2AX in p53+/+ cells that underwent senescence while the upregulation of ϒH2AX was detected in p53−/− cells treated with DPI and was correlated with DNA fragmentation. Recently another activity of DPI of great anticancer potential has been discovered. Namely, Ozsvari et al. (2017) [50] have shown that DPI, already at very low doses, inhibits mitochondrial metabolism and reduces mitochondrial driven ATP production in a reversible manner. The culture of MCF-7 breast cancer cells in the presence of DPI for 5 days led to significant reduction of cancer stem cells (CSCs) [50]. Still, the molecular mechanism responsible for selected activity of DPI towards CSCs remains undiscovered and needs further investigation. However, we might speculate that both, senescence induction and CSCs depletion induced by DPI, might be responsible for the anticancer potential of this inhibitor.

Importantly, cancer cell senescence remains a field of controversy. There are proofs including those from our laboratory, that cancer cells can escape senescence and regain proliferation potential [51,52,53]. Thus, senescent cancer cell can be perceived as potentially dangerous, cancer dormant cell that might, under certain circumstances, lead to tumor recurrence. Fortunately, senescent cells might be selectively eliminated by compounds called senolytics, which target prosurvival as well as antiapoptotic pathways active in senescent cells [54,55,56]. So far, a number of studies have shown that senolytics are effective in treatment of age-related diseases. Thus, it is reasonable to expect that combination of senescence-inducing drugs with senolytics might be useful in developing the most efficient anticancer strategies.

## 5. Conclusions

To summarize, we have demonstrated that the inhibitory potential of DPI on cancer cell growth results from senescence induction due to decreased ROS level. This prosenescence activity depends, to a certain extent, on wild-type p53 expression while p53-deficient cells are more susceptible to apoptosis induction. In any case (p53+/+ and p53−/− cancer cells), a long-lasting effect of flavoenzymes inhibition leading to restriction of cancer cell growth might be observed which supports the therapeutic potential of this compound.
